# Cost-Effective Resources for Computing Approximation Queries in Mobile Cloud Computing Infrastructure

**DOI:** 10.3390/s23177416

**Published:** 2023-08-25

**Authors:** Arun Kumar Sangaiah, Amir Javadpour, Pedro Pinto, Haruna Chiroma, Lubna A. Gabralla

**Affiliations:** 1 International Graduate School of Artificial Intelligence, National Yunlin University of Science and Technology, Douliou 64002, Taiwan; 2 Department of Electrical and Computer Engineering, Lebanese American University, Byblos 1102-2801, Lebanon; 3 Department of Computer Science and Technology (Cyberspace Security), Harbin Institute of Technology, Shenzhen 150001, China; 4 ADiT-Lab, Electrical and Telecommunications Department, Instituto Politécnico de Viana do Castelo, 4200-319 Porto, Portugal; 5 College of Computer Science and Engineering, University of Hafr Al Batin, Hafar al-Batin 31991, Saudi Arabia; 6 Department of Computer Science and Information Technology, Applied College, Princess Nourah Bint Abdulrahman University, Riyadh 11671, Saudi Arabia

**Keywords:** intelligent technique algorithm, peer to peer, particle optimization, approximation queries, mobile cloud computing

## Abstract

Answering a query through a peer-to-peer database presents one of the greatest challenges due to the high cost and time required to obtain a comprehensive response. Consequently, these systems were primarily designed to handle approximation queries. In our research, the primary objective was to develop an intelligent system capable of responding to approximate set-value inquiries. This paper explores the use of particle optimization to enhance the system’s intelligence. In contrast to previous studies, our proposed method avoids the use of sampling. Despite the utilization of the best sampling methods, there remains a possibility of error, making it difficult to guarantee accuracy. Nonetheless, achieving a certain degree of accuracy is crucial in handling approximate queries. Various factors influence the accuracy of sampling procedures. The results of our studies indicate that the suggested method has demonstrated improvements in terms of the number of queries issued, the number of peers examined, and its execution time, which is significantly faster than the flood approach. Answering queries poses one of the most arduous challenges in peer-to-peer databases, as obtaining a complete answer is both costly and time-consuming. Consequently, approximation queries have been adopted as a solution in these systems. Our research evaluated several methods, including flood algorithms, parallel diffusion algorithms, and ISM algorithms. When it comes to query transmission, the proposed method exhibits superior cost-effectiveness and execution times.

## 1. Introduction

The advancement of distributed technologies, such as grid, peer-to-peer networks, and mobile computing, has revolutionized the landscape of large-scale collaborative applications. Among these distributed technologies, peer-to-peer systems have gained significant attention for their scalability, simplicity, and independence from powerful centralized servers. In peer-to-peer systems, data storage and processing can occur autonomously across the network, and users can freely join or leave the network at any moment, fostering a highly flexible and dynamic collaborative environment [1,2].

One of the key challenges in mobile cloud computing (MCC) is to ensure safety and security within the mobile environment. With the widespread usage of cloud-based services accessed through mobile devices, the vulnerabilities of mobile networks and potential risks associated with cloud-based data storage demand robust solutions. Recent research trends in MCC focus on addressing these challenges and enhancing the reliability and security of MCC systems. Researchers in the MCC domain are actively exploring energy-efficient strategies to optimize data transmission, resource allocation, and task scheduling to extend the battery life of mobile devices. Improving the quality of service (QoS) in MCC is also a priority, with efforts concentrated on reducing latency, enhancing network reliability, and delivering seamless user experiences. Trust and reputation management mechanisms have emerged as critical components to assess the reliability of cloud service providers and mobile devices in MCC. These mechanisms play a vital role in establishing trust among users and service providers, thereby enhancing the overall safety and security of data transactions and exchanges. The concept of federated learning holds promise in enabling collaborative model training without exposing raw data. This privacy-preserving approach is well-suited for applications in mobile cloud computing, allowing mobile devices to participate in model training while preserving user data privacy. Furthermore, the integration of blockchain technology with mobile cloud computing has become an area of considerable interest. Blockchain’s tamper-resistant data storage and decentralized trust offer potential solutions to enhance security and transparency within mobile cloud environments. Researchers aim to explore practical implementations of blockchain to ensure data integrity and bolster the safety of mobile cloud computing.

In the realm of industrial applications, the Industrial Internet of Things (IIoT) has brought revolutionary changes, including intelligent power quality monitoring for industrial drives. Recent research trends in this domain focus on developing advanced power quality monitoring systems that can efficiently handle and process large volumes of data.

Researchers are integrating edge intelligence and analytics into power quality monitoring systems to enable real-time analysis at the edge, leading to quicker decision making and increased responsiveness. Additionally, the fusion of data from multiple sensors has emerged as a significant area of exploration to gain comprehensive insights into power quality and overall system performance. This holistic approach allows for timely fault detection and diagnosis, enhancing the reliability and efficiency of industrial drive applications. Cloud-based predictive maintenance systems have become invaluable tools in optimizing industrial drive operations. By leveraging historical data and advanced analytics, these systems can predict equipment failures and schedule proactive maintenance, reducing downtime and optimizing maintenance costs. Energy harvesting techniques are actively being studied to improve energy efficiency and power quality in industrial drive applications. The integration of renewable energy sources and energy storage solutions offers promising avenues to enhance power quality while reducing reliance on traditional power grids. As industrial applications become more interconnected, data privacy and security are critical concerns. Researchers are actively working on developing secure communication protocols, encryption methods, and access control mechanisms to safeguard sensitive data and maintain the integrity of power quality information. In peer-to-peer systems, the dynamic behavior, where users can store their information in different parts of the network, has led to the reliance on data replication to provide a substantial volume of available data. Some replication methods are considered static, but sophisticated applications in peer-to-peer systems require more advanced abilities to identify conflicts in updates. Optimistic replication, which allows for asynchronous version updates, enables applications to function seamlessly even in the presence of disconnected or problematic nodes [3,4]. Furthermore, the advancements in distributed technologies, especially peer-to-peer systems, have propelled collaborative applications to new heights. The research focus on ensuring safety in mobile cloud computing and developing intelligent power quality monitoring systems for industrial drives highlights the industry’s commitment to providing secure, efficient, and reliable solutions in the evolving landscape of distributed computing.

### 1.1. Peer-to-Peer System Characteristics

The concept of peer-to-peer processing finds its historical roots in earlier distribution systems such as NNTP and ICP. While the peer-to-peer pattern is not entirely novel, it introduced specific features that distinguish it from traditional client-server models. These characteristics, which were already present to some extent in prior distribution systems, define the fundamental nature of peer-to-peer systems [5,6].

The first notable characteristic is the ad hoc nature of peer-to-peer systems. Unlike centralized architectures with fixed servers, peer-to-peer networks allow members to join or leave the system at will. This dynamic environment results in a constantly changing network topology. To ensure smooth operations and effective collaboration, peer-to-peer systems require robust organization and coordination mechanisms to handle the continuous fluctuations in membership and network structure.

Secondly, peer-to-peer systems rely heavily on the collective resources of participating members, each of whom typically possesses limited capacity and capabilities. Individual nodes may experience failures due to factors, such as limited processing power, bandwidth, or intermittent connectivity. Consequently, it becomes crucial to distribute the workload efficiently and to maintain a balance of tasks among peers to ensure system stability and optimal performance.

The peer-to-peer processing pattern is not entirely novel. Many prior distribution systems, such as NNTP (Network News Transfer Protocol) and ICP (Internet Cache Protocol), followed a peer-to-peer pattern, although typical peer-to-peer systems replaced a type of distribution system with specified features. These characteristics were, of course, present in prior distribution systems, albeit to varied degrees. The following are some of the characteristics of peer-to-peer systems [5,6].

#### 1.1.1. Ad-Hoc

Members join and leave the system at will, and, therefore, the number and position of active members, as well as the network topology, are always changing. Ad hoc nature requires the organization of peer-to-peer systems. One of the fundamental characteristics of peer-to-peer systems is their ad hoc nature. In such systems, members have the freedom to join or leave the network at will, leading to a continuously changing network topology. This dynamic environment presents challenges in terms of organizing and maintaining the system effectively. Coordination mechanisms are necessary to ensure seamless communication, resource sharing, and efficient collaboration among peers, despite the constantly fluctuating membership.

#### 1.1.2. Limited Capacity and Reliance on Members

According to survey results, members do not have the ability to be a server; they have limited capabilities and frequently fail. Because of the members’ limited working ability, it is necessary to divide the burden and create a balance among the participating members. Unlike traditional client-server models where powerful servers handle most of the tasks, peer-to-peer systems distribute functionalities among individual nodes. As a consequence, each member typically possesses limited capabilities, and they may experience failures due to factors like limited processing power, bandwidth, or network connectivity. To maintain system stability and performance, peer-to-peer systems must divide the workload and balance responsibilities effectively among participating members.

### 1.2. Advantages of Peer-to-Peer Systems

Despite the challenges posed by the dynamic and resource-constrained nature of peer-to-peer systems, they offer numerous advantages over traditional client-server architectures [7,8].

One of the primary advantages lies in the enhanced scalability of peer-to-peer systems. In centralized architectures, the scalability is often tied to the capacity of the central server. However, peer-to-peer systems can scale more efficiently due to their decentralized nature. With each node acting as both a client and a server, direct communication and resource sharing among peers enable the system to grow in power and resilience as the number of nodes increases.

Moreover, the cost efficiency of peer-to-peer architectures is another significant advantage. Traditional client-server setups may require expensive infrastructure and maintenance costs for powerful central servers. In contrast, peer-to-peer systems distribute the computational and storage burden across participating nodes, effectively sharing the costs among users. For example, in file-sharing systems, the collective storage space provided by all members eliminates the need for costly centralized servers.

Another key benefit lies in the improved scalability and reliability achieved by peer-to-peer systems. Despite lacking a centralized resource, innovative distributed algorithms and techniques enable Peer-to-Peer (P2P) network to exhibit high levels of fault tolerance and robustness. Such adaptability allows the system to handle node failures and adapt to varying network conditions effectively.

The decentralized nature of peer-to-peer systems also grants users increased autonomy in their interactions. Users are not solely dependent on a central server for data access or communication. Instead, they can directly interact with other peers, enabling more direct and efficient sharing of resources. This autonomy can lead to faster content delivery and more responsive interactions.

Furthermore, peer-to-peer systems offer a higher level of user anonymity compared to centralized architectures. In traditional client-server systems, users must reveal their identities to the central server for various operations. In contrast, peer-to-peer interactions occur directly among peers, allowing users to avoid divulging personal information to centralized authorities. This increased privacy and security make peer-to-peer systems desirable for applications where anonymity is crucial.

Computers that engage in a peer-to-peer system often belong to reasonable, independent members; they may choose to share data, quit the system, or send a query, for example. These decisions are not always in line with the system’s aims, and this conflict of interest might undermine the system’s overall growth and efficiency. Therefore, when creating peer-to-peer protocols, participants’ reasoning must be taken into account. Some of the advantages of peer-to-peer systems are as follows [7,8]:

- These systems enhance the system’s scalability by reducing the system’s reliance on centralized management.
- Because nodes communicate directly with one another, we won’t require ca costly structure to communicate with and manage the nodes.
- Because of its great scalability, it will be possible to increase the number of system nodes, thereby expanding the system’s available resources, and a powerful system will thus be formed.

#### 1.2.1. Split and Reduce Costs

The server will have to spend a lot of money to set up a centralized system that can support several clients. Peer-to-peer architecture can assist in spreading this cost among all members. In a file sharing system, for example, the required space will be provided by all members. One of the primary advantages of peer-to-peer systems is their enhanced scalability. In traditional client-server models, the scalability of the system relies heavily on the capacity of the central server. However, peer-to-peer systems can scale more efficiently because each node can act as both a client and a server. This decentralized approach enables direct communication and resource sharing between peers, allowing the system to grow more powerful and robust as the number of nodes increases. Peer-to-peer architecture can significantly reduce infrastructure costs. In client–server systems, setting up and maintaining powerful centralized servers can be expensive. In contrast, peer-to-peer systems distribute the computational and storage burden across the participating nodes, sharing the costs among the users. For example, in a file-sharing system, the required storage space can be provided collectively by all members, reducing the need for expensive centralized servers.

#### 1.2.2. Improve Scalability and Reliability

Because of the lack of a powerful central resource, one of the major tasks is to improve the system’s scalability and reliability, which leads to algorithmic breakthroughs in this field. Despite lacking a centralized powerhouse, peer-to-peer systems have shown remarkable advancements in scalability and reliability. By leveraging the collective resources of multiple nodes, P2P networks can achieve high levels of fault tolerance and robustness. Innovations in distributed algorithms and techniques have enabled peer-to-peer systems to handle node failures and adapt to varying network conditions effectively.

#### 1.2.3. Increase Autonomy

In many situations, users of a distributed network are reluctant to rely on a centralized server because doing so limits them. In the case of file sharing programmers, for example, users can directly download each other’s files rather than relying on a centralized server that may or may not enable them to receive the file. Peer-to-peer systems grant users increased autonomy and flexibility in their interactions. Users are not solely dependent on a central server for data access or communication. Instead, they can directly interact with other peers in the network, enabling more direct and efficient sharing of resources. This autonomy can lead to more efficient content delivery and faster response times.

#### 1.2.4. Anonymous

This term becomes reliant on the same autonomy. Users may not want any other user or server to have access to their system’s information. Anonymity cannot be guaranteed while employing a central server since the server must be able to identify the client, for example, using its URL. Because procedures are performed locally in a peer-to-peer architecture, users can avoid disclosing personal information to others. The decentralized nature of peer-to-peer systems often provides a higher level of user anonymity compared to centralized architectures. In traditional client–server systems, users must identify themselves to the central server for various operations. In contrast, peer-to-peer interactions occur directly among peers, allowing users to avoid revealing personal information to central authorities. This increased privacy and security make peer-to-peer systems attractive for applications where anonymity is crucial.

#### 1.2.5. Model of Software Architecture

Participants in a peer-to-peer system build a covering network among themselves. A logical channel can be established between two members in this cover without a direct physical link between them. This cover network outlines the physical network’s details, resulting in the growth of peer-to-peer systems on the Internet. To be eligible for this coverage, members must install special software. The software consists of two major components: a peer-to-peer layer and a peer-to-peer application, with the possibility of an intermediate component. This peer-to-peer layer is in charge of shaping and organizing the coverage, as well as file replacement. Peer-to-peer applications carry out the functions required by system-specific services. The interface includes auxiliary applications (such as identifying desirable members) that can improve peer-to-peer quality and performance [9,10].

### 1.3. The Necessity and Importance of Conducting Research

Peer-to-peer systems have recently received increased interest from the research and industrial communities, owing to their potential to combine the participation of members’ resources in the form of enormous, shared resources for all users. Today, the file-sharing service has attracted millions of users who have exchanged massive volumes of network data. Recent scale investigations have contributed to the expansion of this system. According to these studies, peer-to-peer file sharing consumes more bandwidth than the use of global spreadsheets [6]. Participants in a peer-to-peer system collaborate to provide the desired service. This service could be shared processing, file sharing, shared storage, or data exchange, for example. There should be no centralized authority of controlling, organizing, monitoring, or maintaining the entire system. These functions are instead shared among members who collaborate to share resources, such as memory, processor, network bandwidth, and information. The following are some of the advantages of using a peer-to-peer approach for distributed applications:

- Improve scalability by sharing resources among members and minimizing dependency on a centralized server.
- Cost-effectiveness in terms of utilizing available resources and avoiding the need for costly infrastructure
- Capability to grow by completing all procedures in the final system

For numerous reasons, data replication is critical in distribution networks, particularly peer-to-peer systems. First, replication increases system accessibility by removing defects. Second, it enhances system performance by decreasing communication with superiors, and finally, repetition promotes system scalability by facilitating system growth by establishing acceptable response times. Another issue with data replication is managing updates. A copy management system is one that creates a copy, finds the best copies, and manages and updates the copy in a virtual organization. The data copy strategy’s role is to determine when to copy, where to put the copies, and how to put the best copy. The copy algorithm is in charge of generating the dynamics of new versions. This algorithm must decide which files should be copied and where they should be saved. The copy deletion algorithm is in charge of removing duplicates in order to preserve storage space [11]. To achieve a performance gain, a copy selection method is also required. Copy strategy determines the time and location of copy production. This method is influenced by factors, such as data demand, network status, transmission costs, and storage costs. Replica management is a critical issue in decreasing bandwidth consumption, improving data access, and maintaining data inclusion in large distribution networks. Grand River Mutual (GRM) [12,13] refers to maintaining data integrity across the network, which is especially critical in multi-group distribution systems. GRM, on the other hand, is unsuitable for many applications because too many messages must be exchanged during the copy management process, which decreases the amount of message exchanges required to accomplish the appropriate GRM approach by employing an internal connection structure known as a Distributed Spanning Tree (DST) [14,15]. The DST Ver.1.4.6 software turns the network collaboration into a logical layer structure, resulting in a hierarchical method for copy management. This hierarchical method has been found to increase data access and integration throughout the network, as well as to reduce the amount of data recorded and the number of messages transmitted for each application on the network. Due to the distributed nature of peer-to-peer networks, in which nodes with the same role and capabilities exchange information and services directly with each other, each node must request the transmission of multiple network messages in order to exchange data with another network collaboration, which may result in increased access delays. Data copying is an important and widely used method for lowering network access latency and transmission intensity. Copy management is critical for all forms of distribution schemes due to the documented capabilities and desire for network collaboration. Most techniques of copying data copy it to all nodes, or at least to the beginning and end of a linked search path, to boost accessibility. However, a high number of copies consumes a lot of memory and raises the unit cost of individual nodes. Because of these constraints, there is a greater need for a series of efficient solutions to reduce the number of copies. Many solutions have been presented, and they differ in terms of location, durability, scope, and application.

### 1.4. Hypotheses

Along with technological advancements, peer-to-peer systems are now being regarded for having a suitable platform for shared applications, as well as scalability, simplicity, and the lack of the need for powerful servers. Data storage and processing can be conducted independently in any area of the system in a distributed fashion, and users can join or leave from the network at any moment. Because of these dynamic characteristics, which allow users to store their information in different areas of the system in a dispersed manner, they now rely on a new technique to supply a significant amount of available data to peer-to-peer networks. One strategy to improve access to information is to reduce execution time, bandwidth usage in peer-to-peer networks, and overall system performance by employing an up-to-date and appropriate data management method. This method is known as data replication. It is an efficient method of increasing performance and accessibility by storing many replications in different locations as well as reducing bandwidth consumption and access costs in the operating environment. Transparency in data replication is required. The following hypotheses are briefly stated in this study:

- Peer-to-peer networks are highly significant today, and data replication is one of the primary issues of data management in peer-to-peer networks.
- Dynamic data replication is an effective method for controlling the volume of traffic and the performance of peer-to-peer networks.
- Data replication approaches based on intelligent searches have been used successfully for resource management concerns in cloud computing, grid networks, and other domains.
- In peer-to-peer networks, data replication operations are carried out using intelligent and hybrid processing algorithms.
- Synchronous updating of a copy of data by distinct nodes causes duplicates to diverge and collisions to occur. Dynamic data replication methods are employed for this purpose.

### 1.5. Peer-to-Peer Systems and Research Background

Peer-to-peer systems have evolved as a popular method for distributing massive volumes of data. These networks have grown in popularity in recent years. Peer-to-peer networks are fundamentally distributed systems that lack centralized structure or control. When there are multiple iterations in a system that are all based on the same data source, each change in one of them must be reflected in all iterations in order for the iterations to be compatible. If a source has a large number of iterations, keeping these iterations up to date becomes one of the system’s key difficulties if one of them changes and reduces the system’s performance. Memory limits may not be a relevant consideration in many applications nowadays, owing to the arrival of high-capacity memory, but given that we deal with a huge number of massive data sources, memory issues and restrictions are one of the primary limitations. 

The first category is static iterations, in which the iteration policy is specified from the beginning and is in fact part of the system configuration, i.e., the location of the iterations is specified in the system design stage, and these iterations are located in the specified locations during the implementation stage and, moreover, their location is set and unchanging till the conclusion of system operation. The first category is static iterations, in which the iteration policy is specified from the beginning and is, in fact, part of the system configuration, i.e., the location of the iterations is specified in the system design stage, and these iterations are not only located in the specified locations during the implementation stage but their location is also constant and unchanging till the conclusion of system operation. Changing the topology of the data source network or the pattern of queries will almost certainly not modify this policy. Therefore, the efficiency of the system is severely reduced and resources may not be used properly. For example, the number of system users may be statically set at 50 at the time of policy setting, but after a while, the number of users may reach 500, or initially the number of users in a particular node may exceed the rest of the nodes, but this pattern changes and the traffic of another node increases [16].

As a result, these algorithms are more suited for applications where the user request pattern is fairly constant and highly predictable. If the user request pattern in these systems changes, the only method to modify the system is to redesign and re-implement the system. This is the most significant disadvantage of these approaches. The most significant advantage of these systems is their simplicity of implementation. Table 1 summarizes the presented approaches separately.

In systems where the user request pattern is not constant and changes over time, using static repetition methods is not only ineffective but may also change the present configuration to one of the worst scenarios by changing the user request pattern. Therefore, methods for dynamically determining the optimal configuration of iterations based on the current demands of users are required in such systems. Dynamic replication methods are designed for this purpose, in which the data source automatically replicates as needed owing to changing access patterns and other variables, and places it in the appropriate location to improve access frequency while avoiding cost increases [17]. These approaches continuously monitor the user request pattern and, if it changes, modify the arrangement and configuration of replications to retain the system’s approximate optimal configuration. The process of generating dynamic replication is both centralized and distributed. Versions are created solely at the vertex node in the centralized version, while versions are created at several selected nodes in addition to the vertex node in the distributed version. Dynamic replication approaches outperform static iterative methods because they may make intelligent decisions regarding iteration creation based on information from the p2p network environment [18]. One disadvantage of this strategy is that a central iteration decision maker is necessary in data sources that require the collection of information from all nodes or sites running in a complicated structure. If nodes are added and withdrawn on an irregular basis, the overhead of this central decision maker is compounded. Synchronization is extremely difficult and almost impossible in complicated contexts such as P2P networks when using decentralized approaches. In general, static data replication methods, while simple to construct, are not feasible in practice; on the other hand, dynamic data replication is an optimization strategy that reduces average execution time. Now, despite all the research on dynamic data replication in p2p environments, the following challenges remain. High execution time on peer-to-peer sites, high file access delays, absence of combined use of iterative algorithms and scheduling in the majority of proposed techniques, limited number and capacity of available storage resources, and inefficient data access [19]. Replica techniques must have the ability to improve one or more optimization parameters of iterative algorithms. The optimization parameters of iterative data algorithms improve system performance, and each proposed data replication approach must do at least one of the following: reduce access latency, bandwidth usage, maintenance costs, strategically placing replications, and downtime, create load balancing, increase fault tolerance, quality assurance, and make the most use of available storage resources. Search methods in data iteration algorithms can be categorized into conscious or blind. In blind search, nodes do not hold any information about the location of documents, but in conscious search, there is a distributed or centralized directory service that aids in the search for the requested items. The basic idea behind these search algorithms is to decrease the amount of nodes that each query receives and evaluates. This will be processed, and fewer results will be returned [20]. The proposed architecture, known as eestore [21], introduces a principled distribution list with a repeat layer in the middle and a transaction management layer at the top layer. The authors of this reference presented a method called self-scaling iteration, which includes features including automatic data segmentation, load balancing, efficient query range, transactional access, and compatibility with agreed-upon replication and multi-copy concurrency management. They presented the inclusion of effective masses in an ordered distributed table in [22,23]. The planning step comes before the actual degrees in this method. This method’s characteristics include the employment of parallel clusters, balance between interpolation, and the decrease in partition transfer costs while developing operational capacity. The method of partitioning and iteration on the load axis, known as division, is provided in the reference. The approach is divided into two stages: load-oriented and division and iteration based on the graph, distribution and validation. In the article [24], databases are presented as a service with specific purposes that aim to strengthen large organizations’ data management capability. This system, which has numerous dedicated storage engines and high access due to transparent replication and automatic partitioning, has become highly scalable and balanced by allowing for regular distributed transactions. The authors present a method in the reference that separates large databases into node sets and converts a large number of small, independent databases into multi-tenant databases. Thus, by improving transaction and fault tolerance, they have provided a very flexible method. The network distance and load balancing of the nodes are measured in this approach, and an algorithm called Paxos is employed for this purpose. The data replication technique in the article [25] is the way of integrating the file collection with the FIRE method, which performs better than the LRU and LFU methods by enhancing efficiency and decreasing data access time. The DRS data replication approach is used in a peer-to-peer network environment to improve data access, which reduces network latency and detects changes in the access algorithm, speeds up data access, reduces data transfer rates over long distances, and boosts efficiency and decreases bandwidth. In [26], the authors used a dynamic table with indexing by presenting an intelligent method called RPAT. This discovery approach is based on evaluating the similarity of data sources on the path of requesting and service nodes, and it may be utilized in decentralized networks and dynamic algorithms due to the ability to establish and update rules. Researchers introduce OPRA, an intelligent and discovering technique, in [27], which compensates for the time delay caused by node requests using the pointer iteration method. This algorithm’s methodology is greedy, and it aims to reduce the retrieval rate of the query by using a pointer table. The researchers’ notion in [28] is that if we allow nodes to reply to queries on behalf of other nodes, we can still reduce the number of nodes that process the query to respond to most queries without relying on the capabilities of multiple nodes. This will decelerate network access to data sources. Current lookup algorithms aim for efficient bandwidth and the discovery of unique objects via these networks. In these systems, the respondent node may be aware of the query’s source address and may establish a temporary connection to the source (to convey the response message). Although this method consumes more bandwidth than the previous ones, it provides anonymity for the query source and protects it from being targeted by connection requests. When a node receives a query message, it must determine whether to forward it to its other neighbors or destroy it. The routing policy decides whether the query is sent and to whom. This strategy is implemented by having a search source that queries messages exclusively to a subset of neighbors who select nodes that produce quality results. The lookup approaches considered by researchers nowadays are highly distinct and have numerous capacities in the field of intelligence, some of which are discussed following. Data Grid is a distributed environment that deals with high-volume centralized data applications.

**Table 1:** An overview of the research background and related methods.

[29]	HRI	Try to select the best super node to refer to prevent duplication and fetching of queries between nodes in an area [30].
[31]	An intelligent fuzzy search method based on clustering topology	The existence of a cluster means that when a request for a particular object reaches the cluster, the request can be sent to the part of the cluster that has the best chance of finding the desired file and source [32].
[33]	Random-walk algorithm is used	The Random-walk algorithm is used to improve the response time [34]. In order to solve the flooding problem that causes problems, such as heavy traffic on the network
[35]	object replacement	In this method, distributing a file within the network and repeating it between different nodes reduces the search pressure and also the time to find that file within the network [12].
[36]	From a data structure	Intelligent strategies based on the Digistra method and Storage Strategy Query categorizations classify the output results as approximate or accurate [37].
[38]	Maintain the node list	Each node holds a list of other nodes that are connected to a network. In this list, nodes that are directly connected to the node are referred to as neighboring nodes, and the number of these neighbors is considered a degree for this node.
[39]	Schedule called CSS	Divides the optimization problem into several pieces and distributes them over the network so that it can be used to monitor lost information and retrieve missing pieces of information from neighboring peers [40].
[28]	Query	The search techniques considered by researchers today are very different and have many capabilities in the field of intelligence, some of which are discussed below. Data Grid is a distributed environment that deals with high-volume centralized data applications.
[27]	OPRA	Compensates for time lags caused by node queries by pointing method
[26]	RPAT	This detection method is based on calculating the similarity of data sources on the path of request and service nodes, which can be used in decentralized networks and dynamic algorithms due to the ability to create rules and optimize them.
[25]	FIRE	In a peer-to-peer network environment, the DRS data replication strategy is used to improve data access, which reduces network latency and detects access algorithm changes, speeds up data access, reduces long-distance data transfers, and increases performance and reduces bandwidth.
[24]	Data management for large organizations	This system has several dedicated storage engines and has high access through transparent duplication and automatic partitioning.
[23]	Insert effective mass in a table	In this method, the planning phase is before the actual degrees. The use of parallel clusters, balance between interpolation and reduction of partition transfer costs with the development of operational capacity are the features of this method.
[21]	Eestore	A principled distribution list with a duplication layer in the middle and a transaction management layer in the top layer.
[20]	Blind lookup	Nodes do not hold any information about the location of documents, while in conscious methods, there is a distributed or centralized directory service that helps to search for the requested objects.

The algorithm [39] contains a scheduler called CSS that takes into account the number of tasks waiting in line, the location of the data necessary for the tasks, and the processing capabilities of the nodes. The optimization problem is separated into several portions and distributed over the network in this article in order to monitor the missing information and retrieve the missing patch information from the neighboring peer. Protocols demand a delay between the time it takes to create packets at the source and the time it takes to broadcast on the network in this reference. The authors of [38] developed an approach in which each node in a network maintains a list of other nodes that are connected to it. Nodes that are directly related to the node are referred to as neighbor nodes in this list, and the number of these neighbors is regarded a degree for this node. As the number of nodes increases, the maximum size of the path from one node to another decreases; however, this strategy increases the storage space in each node. Although this method use TTL for each message to avoid requiring each node to store a huge volume of messages, the TileLife problems make the method inefficient. Intelligent techniques based on the Digistra method and query classifications are used in [36] to classify the storage strategy of output results into approximate and precise methods. The suggested technique employs a distinct data structure. The new method employs a tree data structure with less search space, which decreases runtime and memory requirements. Furthermore, the proposed solution accounts for the CPU constraint by raising the data entry rate, which increases system burden. The system recognizes this circumstance automatically and reduces some extra load. Using a statistical technique, the error rate of the final findings is assured to be confined to the amount of error predetermined by the user. The authors of [35] also propose an object replacement mechanism for selecting the location of a node in the network so that it has a higher probability of being found. Distributing a file throughout the network and repeating it between multiple nodes minimizes the lookup pressure as well as the time required to locate that file within the network. Duplicate subgraphs are subgraphs that are frequently observed in path nodes on a graph and provide the most repetition with the least support while exploring the pattern of the graph with k using the greedy local search technique. In [33], in order to solve the flooding problem that causes problems such as heavy traffic in the network if the TTL parameters are not well defined, the Random-walk algorithm is used in which a node is randomly select one of its neighbors and then transmit its request message. The node neighbor also selects another neighbor at random and sends the message again. This step is repeated till the TTL message is finished. If the initial neighbor does not react to the desired message, the primary node will send the request to another random neighbor, and this procedure will be repeated until the source is discovered or a fault is encountered. To improve response time, we can send several requests in one unit on the network using Random Walk. The authors of [31] proposed an intelligent fuzzy search approach based on clustering topology in which navigation can be performed and used based on the geographical distance of nodes or their identical features in their shared resources. The existence of a cluster means that when a request for a particular object reaches the cluster, the request can be sent to the part of the cluster that has the best chance of finding the file and the source, because the source and file identifiers are amazingly near to the identification of the cluster’s certain series of nodes. The authors of [29] attempt to select the best super node to refer to by employing a method that employs the Hop-Count Routing Indices (HRI) algorithm to prevent duplication and fetching of queries between nodes in an area, claiming that their random routing has solved the problems of random walk and flooding. 

## 2. Research Statement and Proposed Methods

Peer-to-peer architecture was initially conceived as a network architecture for sharing computer system resources. Peer-to-peer database systems include infrastructure that is a peer-to-peer network, and the peer-to-peer network that underlies these systems is effective in many aspects of the system, including query processing and optimization. Participants in a peer-to-peer system collaborate to provide the desired service. This service could be shared processing, file sharing, shared storage, or data exchange, for example. There should be no centralized body in charge of controlling, organizing, monitoring, or maintaining the entire system. These functions are instead shared among members, who collaborate to share resources, such as memory, CPU, network bandwidth, and information. Due to the distributed nature of peer-to-peer networks, in which nodes with the same role and capabilities exchange information and services directly with each other, multiple network messages must be requested for each node to exchange data with another partner on the network, resulting in increased access delays. Data copying is an important and widely used method for lowering network access latency and transmission intensity. Copy control is critical for all forms of distribution programs due to the documented capabilities and demand for partner networks. 

### 2.1. Intelligent Object Search

The search algorithms presented in this study are intended to be efficient in terms of bandwidth and the discovery of required data sources via peer-to-peer networks. Methods of searching can be classified consciously or blindly. In blind search, nodes have no knowledge of the location of documents, but in conscious techniques, there is a distributed or centralized directory service that aids in the search for the requested items. The basic idea behind these search algorithms is to limit the number of nodes involved in receiving and processing each query. Intelligent and exploratory strategies will be effective only when the majority of queries can be answered by a small number of nodes. The innovative idea presented in this study is to provide a method that allows nodes to respond to queries from other nodes, reducing the number of nodes that process the query and, as a result, the need for and reliance on multi-node capabilities in the network. When a user issues a query, the corresponding node is the query’s origin. The query message may be sent by the source node S to any number of its neighbors. The routing policy sets the number of neighbors and which neighbor receives the query. When a node receives a query message, the query is processed throughout the node’s local set. If a result is found in that node, it will send a distinct response message to the query source.

We can create some exploratory methods to help choose the best neighbor to send a query. Some of the exploratory methods are:

- Choose the neighbor who provided the most results for earlier queries.
- Choose a neighbor who returns reply messages with the fewest stations on average. Fewer stations may mean that this neighbor is closer to nodes that have useful data.
- Choose the neighbor who has sent the most messages (of any type) since our client connected with the neighbor. The enormous quantity of messages suggests that the neighbor is stable, implying that we have been connected to it for a long time and that it can handle a significant flow of communications.
- Choose the neighbor who has the shortest message queue. The long message queue indicates that the neighbor level has reached saturation or that the neighbor has died.

### 2.2. Network Structure in a Peer-to-Peer System

**Figure 1:**
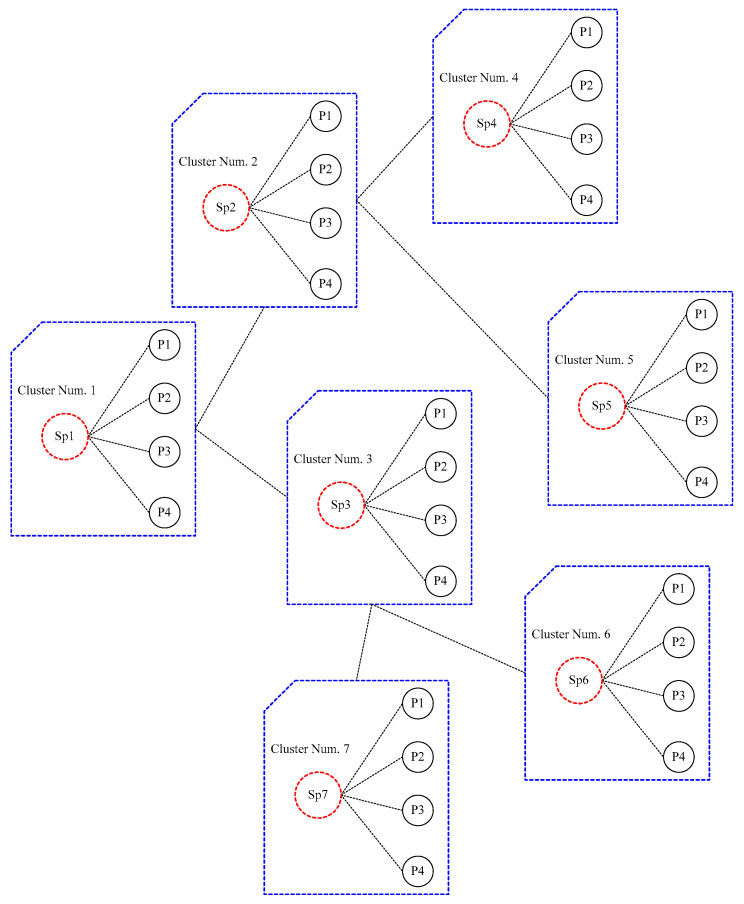
Super-peer network model.

The proposed network topology in this paper is based on the supergroup. Nodes in this network are classified into two types: super peers and client peers. Each super peer is linked to a group of client peers. The super peer serves as a centralized server for a number of peers in the cluster, and communication is handled by these lead nodes. The architecture used in this article is depicted in the Figure 1.

The Figure 1 depicts a peer-to-peer network with each cluster consisting of a leader and a client. Each cluster is the same size as the number of nodes in the cluster (client peers and super peers). Through super peers, clusters can communicate with one another in network coverage. When a client enters the system, it transmits data information to its cluster header, which is then added to the header indexes. When a node is removed from the network, the same thing happens. Clients submit their updates to the cluster header when they change information (for example, registering, deleting, or modifying an item).

### 2.3. The Proposed Structure

The approach presented in this study is to first identify the same of all similar queries in the current query using the query similarity criterion, and then select a set of neighbours that provided the most results for these queries. If there is a collision, the query will consider the path back to the requester and update the local indexes. This technique, which achieves high accuracy, can exchange knowledge while imposing no overhead on node entry and exit. In other words, the creation of its message is massive and only grows with time (when knowledge is spread over the nodes). This algorithm is incompatible with deleting objects or leaving peers because it does not use negative feedback from searches and sending is based on ranking. Finally, its accuracy is strongly reliant on the assumption that nodes be assigned to specific documents. The purpose of this strategy is to minimize the number of messages exchanged for each request and the number of nodes analyzed, so the request message is delivered to those nodes rather than all nodes.

Finally, its precision is strongly reliant on the assumption that nodes be assigned to specific documents. In this method, the purpose is to decrease the number of messages exchanged for each request and the number of nodes examined, so the request message is not sent to all nodes, but only to those nodes that are most likely to respond to the current request, and, for this purpose, the record-keeping mechanism, which is used by each node to record the data of its neighbors, is used. It keeps track of neighbors’ responses to recent requests. The answer T, for example, holds the most recent request for each neighbor.

### 2.4. How to Select a Peer to Transmit Information

When the memory is full, the LRU (Unified Modeling Language) method is utilized to replace the data. In the ranking system, the rank which is defined by each node p to define its neighbors’ rank based on their history, and based on this ranking, the request is forwarded to the neighbors who are more likely to respond to it. 

The following formula is used for ranking, in which the Cost function calculates the similarity of the latest request with prior requests and the s function indicates the number of responses returned by that specific neighbor to the request. The relevance of the similarity of the two requests is indicated by parameter a, and the larger the value, the better the rating of the neighbor who responds to the same request. This strategy is beneficial when the nodes include information about a specific topic:

$$RRpl\left(P_{i}.q\right)=\varepsilon Cost{\left(q_{i}.q\right)}^{\alpha }*S\left(P_{i}.q_{k}\right)$$

One problem with this strategy is that if a new node contains very relevant data, the request message will not reach it and will instead travel to the prior nodes because this new node has no history of rising in the rankings. One method is to choose additional nodes at random in addition to the nodes selected by the ranking. We recommend using the local indexing strategy. The range of each node’s depth stations is held by node n, the data index of each node in the local index technique. The index radius is denoted by the symbol r. The node that receives the query message can process it from any node within its r station depth range. As a result, multiple node sets can be searched by query processing in a small number of nodes, resulting in a high degree of satisfaction and results while keeping costs low.

### 2.5. Local Indexing Policy

The distinction between local index politics and repetitive deep learning duplication policy is that in the deep learning duplication technique, the depths of the policy specify the depths at which the repeats should terminate, and nodes of all depths process the query. In order for the local index approach to work as well as the first depth D level search, the local index policy’s final depth must also be set to D-r. When a node departs the network or dies, the relevant metadata is deleted by other nodes that index the set of that node after a certain length of time. Each node indexes the files stored in all nodes within a radius of r and can respond to queries from all of them. The first level search method does a search, but only the nodes available to the requester are processed at particular search depths. To keep overhead to a minimum, the station distance between two consecutive depths should be 2r + 1. Because each connected node indexes numerous peers, the accuracy of this technique is expected to be high and the collisions to be very low.

### 2.6. Routing Indices

The Routing Index (RI) is used in P2P networks to improve query routing and to minimize flooding. This index organizes the nearby nodes’ resource information and, depending on this information, delivers the existing query to the neighboring peers. In this study, we employ the Hop-Count Routing Index (HRI) idea for each peer, which counts the number of nodes required to reach a base level. This index is created as a M × N table, where M is the number of peer neighbors and N represents the maximum number of steps. This index is created as a M × N table, where M is the number of peer neighbors and N represents the maximum number of steps. The number of data elements that can be accessed via a peer neighbor is represented by the nth place in the mth row of this matrix. When a new partner enters the network, it provides data information to all of its neighbors. As a result, the data in the peers has been updated. 

### 2.7. How to Conduct a Client Queries

When a client requests a resource (file), it sends a query message to the local super peer. This query is executed locally, and the results are presented. If a response is available, it sends a successful message with the IDs of the responding nodes to the requesting node. Otherwise, the local node selects the best super peer for the neighbor based on the number of routing indexes and sends a copy of the query to the chosen neighbor. When a source that fits the criteria indicated in the query is located, a queryHit message is generated and sent to the query node via the same return path. This technique is repeated until all neighbors have been contacted or examined.

### 2.8. Search Algorithm

If we consider the network structure shown in Figure 1, the search procedure within the peer-to-peer network will be as follows. When node P1 in cluster 1 requires a resource (a file), it makes a request to the cluster’s head (cluster 1). Cluster 1 (SP1) then looks for the desired cluster. If acceptable requested resources are detected, the requester will be supplied the node address of the owner of the requested resources. Otherwise, the cluster in Cluster #1 sends a request to find the best neighbor based on HRI data. For example, in this case, we will assume that Cluster 2 is better than Cluster 3. As a result, the cluster head in Cluster 2 initiates a local search. If it receives no responses, it forwards the request to its best neighbor (for example Cluster 4). If the result is in Cluster 4, the queryHit value from the node containing the requested file is returned. Otherwise, the query will be returned unanswered, and the Cluster 2 header will route it to the second best neighbor (cluster 6). In general, if the Cluster header 2 lacks the necessary resources and, after reviewing all of its neighbors, is unable to discover an answer to the given query, Cluster header 2 returns the query to Cluster 1. As a result, at this point, the Cluster head 1 forwards the received query to its second best neighbor (Cluster 3). The pseudo-code Algorithm 1 below demonstrates how to run this search method.
**Algorithm 1:** Pseudo-algorithm The proposed method for source search.FUNCTION MatchQueryLocalResource(query)   localResource = NULL   // Logic to match the query with the local resources in the cluster   // …   RETURN localResource FUNCTION FindNextBestNeighbor(query, toTry)   nextBestNeighbor = NULL   // Logic to find the next best neighbor using HRI (Hybrid Routing Index)  // …  RETURN nextBestNeighbor FUNCTION ForwardQueryToRecipient(query, recipient)  // Logic to forward the query to the recipient   // …  // …  // …  // Ensure the query is forwarded to the recipient  PRINT “Forwarding query to recipient:” + recipient FUNCTION SendResponseToRequester(query)  // Logic to send the response back to the requester   // …   // …  // Ensure the response is sent back to the requester  PRINT “Sending response to requester” FUNCTION MainAlgorithm(requests)  FOR EACH query IN requests    localResource = MatchQueryLocalResource(query)    IF localResource IS NULL THEN      nextBestNeighbor = FindNextBestNeighbor(query, toTry)      IF nextBestNeighbor IS NULL THEN         recipient = Sender(query)       ELSE        recipient = nextBestNeighbor       END IF      ForwardQueryToRecipient(query, recipient)    ELSE      SendResponseToRequester(query)    END IF END FOR

### 2.9. In-Depth Explanation of the Proposed Method

The paper introduces an intelligent object search method in peer-to-peer (P2P) systems using particle optimization. Particle optimization, also known as particle swarm optimization (PSO), is a metaheuristic optimization algorithm inspired by the social behavior of bird flocking or fish schooling. It is commonly used to solve optimization problems, and in this context, it aims to improve the efficiency of object search within the P2P network. The goal of the intelligent object search method is to efficiently discover required data sources through P2P networks while minimizing bandwidth consumption. The search algorithms are designed to strike a balance between accuracy and the number of nodes involved in processing each query. The search methods can be classified into two types: conscious search and blind search. In blind search, nodes have no prior knowledge of the location of the desired documents. On the other hand, conscious search methods involve a distributed or centralized directory service that aids in locating the requested items. The core idea behind these search algorithms is to limit the number of nodes involved in processing each query. Intelligent and exploratory strategies are employed, assuming that the majority of queries can be answered by a small number of nodes. By doing so, the method aims to reduce access delays and improve the overall efficiency of the object search process. To achieve this, the paper proposes a method where nodes in the P2P network can respond to queries from other nodes, effectively reducing the number of nodes that process the query and thereby reducing the reliance on multi-node capabilities in the network. When a user issues a query, the corresponding node becomes the origin of the query. The proposed intelligent object search method considers various factors in selecting the best neighbor node to send the query to. Some of the exploratory methods employed are:

- Choosing the Neighbor with Most Results: Selecting a neighbor who has provided the most results for previous queries.
- Choosing the Neighbor with Fewest Stations on Average: Preferring a neighbor who returns reply messages with the fewest stations on average. Fewer stations may indicate that the neighbor is closer to nodes with useful data.
- Choosing the Neighbor with the Most Messages Sent: Prioritizing a neighbor who has sent the most messages (of any type) since connecting with the current node. A higher number of messages suggests stability and an ability to handle significant communication.
- Choosing the Neighbor with Shortest Message Queue: Preferring a neighbor with the shortest message queue. A long message queue might indicate that the neighbor has reached saturation or is no longer active.

The proposed method also employs a ranking system where each node defines its neighbors’ ranks based on their history. The ranking system helps forward the request to neighbors who are more likely to have the desired data, reducing the number of messages exchanged for each query. Additionally, the paper introduces the concept of a “local indexing policy”. Each node maintains an index of files stored in all nodes within a certain radius. This allows query processing to be restricted to a smaller set of nodes, thereby reducing overhead and increasing search efficiency. The Hop-Count Routing Index (HRI) is also used to improve query routing and minimize flooding in the P2P network. The HRI idea involves counting the number of nodes required to reach a base level, and the index is organized as an M × N table, where M is the number of peer neighbors and N represents the maximum number of steps. To conduct a client query, the client sends a query message to the local super peer. The super peer handles the query locally, and if it has the desired data, it responds with the results. If not, it selects the best super peer based on the Hop-Count Routing Index and forwards a copy of the query to the chosen neighbor. This process continues until the query is answered or all neighbors have been contacted. Overall, the intelligent object search method uses a combination of exploratory strategies, ranking mechanisms, local indexing, and the Hop-Count Routing Index in order to improve the efficiency of object search in P2P systems. However, the paper could provide more in-depth details about the specific implementation of the particle optimization technique, how it interacts with the search algorithms, and its impact on the overall performance of the intelligent object search method to allow other researchers to better understand and replicate the results.

## 3. Simulation and Results

In this study, a peer-to-peer system is developed to provide the desired service, which is to find an answer to a question or service among peers. This service could include file sharing, shared storage, or data exchange. The purpose of this research is to find an optimal or near optimal answer to the query. The following is the procedure for obtaining the best possible answer to a query. We must determine the best path from the source peer. The best approach provides the greatest number of responses by allocating TTL per hop, and this is while we aim to keep the cost-to-response ratio as low as possible in this direction. In other words, the number of hops required to reach an answer is minimized in an optimal path. The following equation illustrates the formal definition of the optimal response to a query:

$$optimal\;Answer={Path}_{Optimal}\;where\;\min\left(\frac{{Cost}_{PathOptimal}}{{Hits}_{PathOptimal}}\right)$$

### 3.1. Simulation Environment

The simulations are conducted in an environment with an Interl^®^ Core ™ i7 CPU @ 2.3 GHz and 6 GB of RAM. The operating system used is Windows 7 Ultimate 64Bit. MATLAB programming language is used to simulate a peer-to-peer system, the relationship between databases and the structuring of data releases.

### 3.2. Initial Values

In this section, we considered four different sources with varied parameters for each of the examples, as shown in the table below. Each parameter has a minimum value that can be incrementally increased to a maximum value. We produced one of the sources at random for each peer using the parameters specified in Table 2 and assigned these sources to the relevant peer. We use the k-means clustering algorithm to cluster the peers based on the resources allocated to each peer.

### 3.3. Simulated Peer-to-Peer System

**Figure 2:**
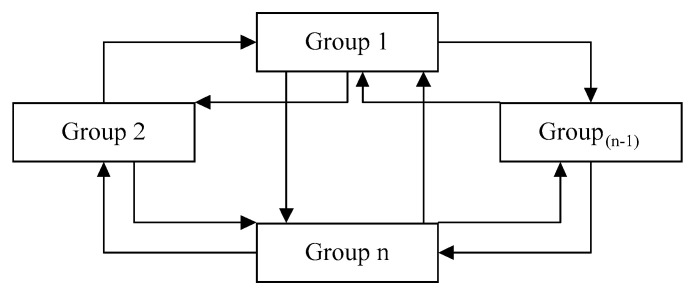
Proposed topology model. All connections between groups are based on the topology of the small world.

The following describes the system environment topology used in this study and the simulation performed for the P2P system. This network topology is represented by the matrix N × N, where N is the number of cluster heads. In this topology, each header is connected to its neighbors by a short-circuit connection, and all of the connections are local. Furthermore, each cluster head is connected to all nodes within the cluster and is topologically related to the nodes inside the cluster like a star. The network size, or, in other words, the initial number of peers, is set to 1024. The equations are classified into 32 groups (using the k-means clustering method). The topology utilized is depicted in the image below. Network topology and content distribution in a dynamic network are also taken into account. The P2P network is presented as a trilogy (peer, link, data). A link is a set of edges that represent all of the network’s links, and data is a dataset in which all of the data are shared. The small world network architecture will be used to model the neighborhood between clusters [30]. This network architecture is represented by lattice N × N. (Figure 2). In this topology, each peer is linked to its neighbors by a short-circuit connection, and all four connections are local. Furthermore, each peer with a D-probability is linked to another peer via a remote connection, where D is the number of hops or the Manhattan distance between these nodes, and where α is also the clustering coefficient, which is considered one.

### 3.4. The Implementation Process

**Figure 3:**
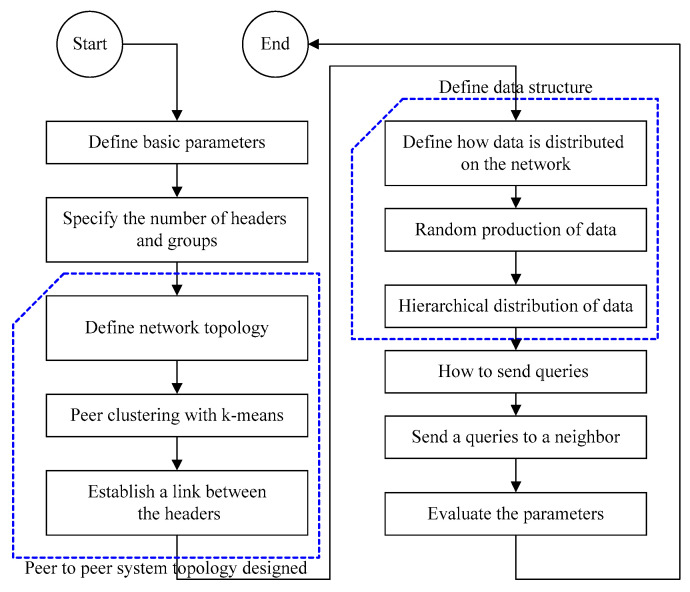
Flowchart of the proposed method’s implementation process.

Peer clustering across different groups was employed in this study in order to create a method for sharing and replicating queries in a peer-to-peer network. Each group has a peer known as the cluster head who is in charge of initiating communication between one group and other groups in the network. The cluster head is responsible for all tasks connected to duplicating queries and sending them to a neighbor who can provide a query response for the asking node. As a result, the cluster head will be chosen from among those with the best sources in each cluster (Figure 3).

### 3.5. A Peer-to-Peer System’s Data Structure

**Figure 4:**
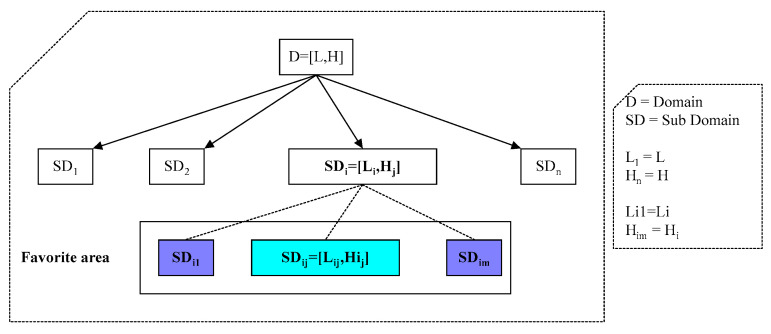
Domain division’s hierarchical structure [41].

The data distribution in this study’s peer-to-peer network is based on the distribution introduced in the study [41]. The domain of the query attribute (the attribute on which the query is issued) is divided into subdomains in this data distribution. This Hierarchical Structure of Domain Division (HSDD) is divided into three levels, with each subdomain at each level equal to the community of its lower subdomains. The lowest level of HSDD consists of 910 subdomains, whereas the second level consists of 177 subdomains. In the third level, each domain in the second level has around five domains. The data structure employed in this paper is depicted in Figure 4.

A total of 30 Tuples are stored in each peer, and the data is not distributed randomly among the peers. Because each peer in a practical situation is interested in specific data, data distribution is based on the idea of the favorite area. Each favorite area comprises a subset of the subdomains. These areas of interest are, in fact, second level subdomains. Each peer’s data is composed of 60% data from one favorite area and 20% data from another favorite area. The rest of the data are assigned at random. Each iteration, each peer asks a question with a 10% probability of being correct. Each query is issued at random on one of the HSSD sheets. Each peer will issue a minimum of zero and a maximum of one question in each iteration, and after completing the execution of all questions in each iteration, the next iteration can start.

### 3.6. Clustering of Peers

The k-means clustering approach is employed in this paper to group peers into similar groups. The parameters shown in Table 3 are used for each peer. The Table 4 depicts the distribution and selection of cluster heads in various clusters. The table of peer indices is maintained and updated in each iteration based on the neighborhoods defined for each cluster. As a result, if a super peer is removed from the network or a new peer is selected as the cluster header, the index table will be updated. The following is how to make changes during program execution.

During program execution, the index table can manage these changes by considering network changes, such as adding a new node or deleting one of the peers from the network and updating the index tables if any of these events occur. When the procedure is performed in the 23rd iteration, a new node is added to the network and identified as the cluster head. This new node cluster head has the index number 35. The index table changes after the 23rd iteration as follows, and it can be noticed that three of the ten cluster heads examined are updating their index tables considering the new cluster head (Table 5).

### 3.7. Values of Parameters

The parameter values used are critical in the particle optimization algorithm and have a significant impact on the program’s efficiency. The Table 6 demonstrates how to set the particle optimization algorithm’s parameters.

### 3.8. Obtaining the Adjacency Matrix

We calculated the cost of crossing each peer as a unit. The best-case situation is to find the answer in a single step. The following findings are produced using the initial values that were considered. We have 32 groups, with 32 peers in each, according to the proposed architecture.

**Figure 5:**
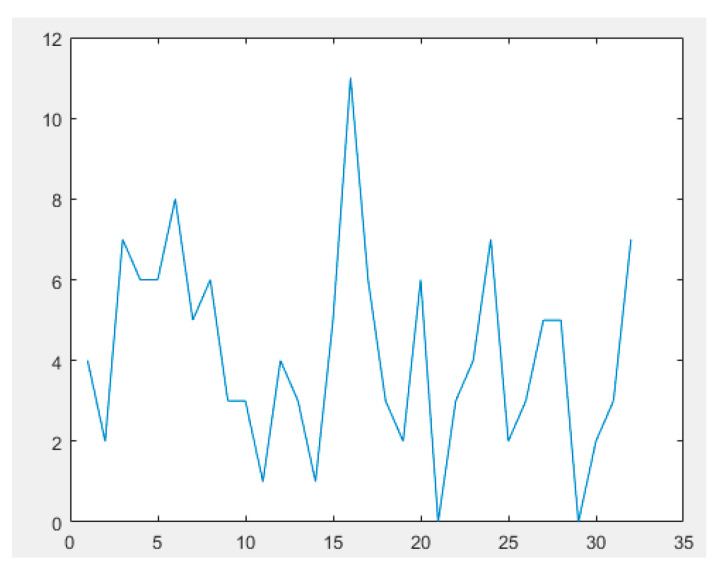
Number of connections between clusters.

In the simulations, peers 1 to 32 are cluster heads, and the remaining peers are in groups. The Table 7 indicates how many communication linkages exist between certain groups out of the 32 available. The proximity matrix stores these connections. Figure 5 diagrammatically depicts the number of connections between all cluster headers.

### 3.9. How to Ask and Respond to Questions

In this study, the question design process is as follows: at the start of each loop, each peer asks a question with a 10% probability. In order to simulate a dynamic environment, a peer with a 10% opportunity is added to or deleted from our group of peers in each loop. If the cluster’s head is removed, the best match in that cluster is allocated as the cluster’s head. Asking a question by any peer involves asking data from one of the network’s peers. The proposed solution of this research is to discover an appropriate path that can be employed to achieve the answer at the lowest cost. Cluster heads disseminate questions over the network, and each peer must contact the cluster head to submit their request. If the desired answer belongs to the same group as the source, the cost is regarded zero. In reality, in this study, we avoided the expense of analyzing data from within each group.

### 3.10. Implement a Flood Algorithm in Query Transmission

**Figure 6:**
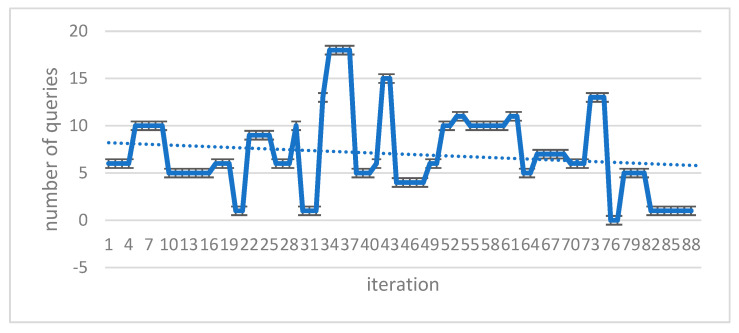
Number of queries submissions in achieving the optimal answer in the first iteration of the flood algorithm.

**Figure 7:**
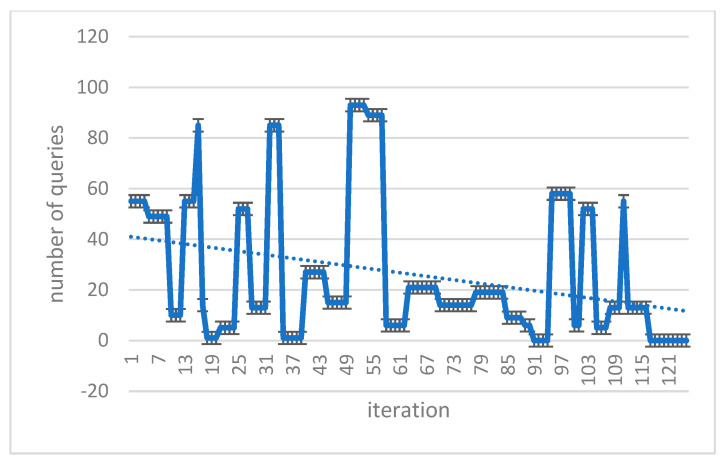
The number of queries submitted to achieve the best answer in the flood algorithm’s hundredth iteration.

In this study, we used the flood algorithm as a reference for comparison to evaluate the efficiency of the proposed method and measure the time required for implementation. The flood algorithm serves as a basic benchmark to assess the performance of the suggested technique. In the flood algorithm, when a peer receives a query, it broadcasts the query to all of its neighboring cluster heads if there is no answer available within its own cluster. This strategy may result in multiple query repetitions and an increase in the program’s execution time. Each peer initiates a query with a 10% probability at the beginning of each loop. The number of queries propagated using the flood algorithm is determined by the neighborhood and adjacency matrix of each cluster head. Due to the broadcasting nature of the flood algorithm, the number of queries sent across the network can grow rapidly, leading to potential inefficiencies in large-scale distributed environments. To compare the proposed method with the flood algorithm, we measure the number of queries issued and the execution time for each approach. The suggested algorithm leverages intelligent search mechanisms, profile-based node selection, and limited message exchanges to optimize query processing and reduce the overall number of queries and execution time (Table 8 and Table 9). The comparison aims to demonstrate the effectiveness and scalability of the proposed method in handling query processing more efficiently than the flood algorithm. By mitigating unnecessary query repetitions and optimizing resource utilization, the suggested algorithm showcases improved performance and reduced execution times, making it a promising solution for large-scale peer-to-peer distributed collaborative applications. The use of the flood algorithm as a reference allows us to assess the advantages and drawbacks of the proposed method in terms of query processing, execution time, and resource utilization. The results obtained through this comparison provide valuable insights into the performance of the suggested algorithm and its potential benefits for real-world peer-to-peer systems with dynamic and varying network conditions (Figure 6 and Figure 7).

### 3.11. The Proposed Method’s Results

**Figure 8:**
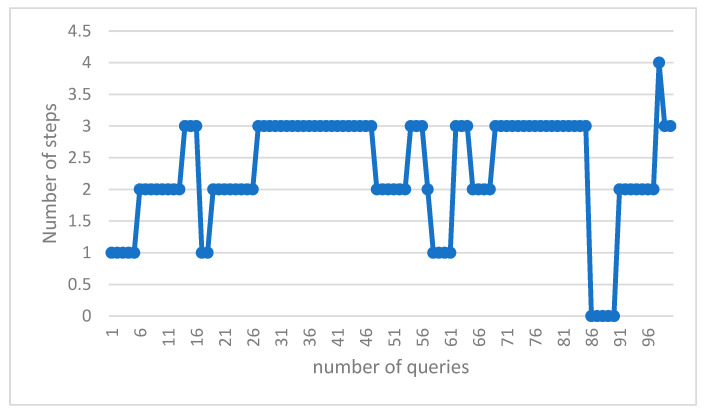
Number of question submissions in achieving the optimal answer in the first iteration.

The diagram below compares the number of questions submitted by peers during the answering process during program execution. The eligibility criterion here is to keep the expense of answering the query to a minimum (Figure 8).

**Figure 9:**
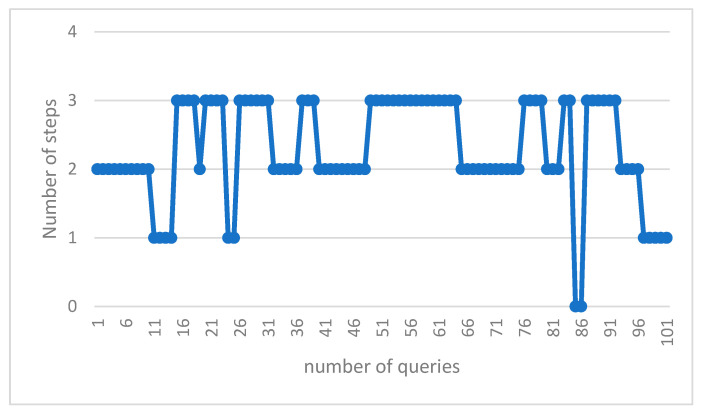
The number of question submissions required to obtain the best response in the hundredth iteration.

The number of scrolls required to achieve the answer in the suggested algorithm is shown in Table 10 and Figure 9. The resulting numbers are related to a few sample queries from all of the particles.

**Figure 10:**
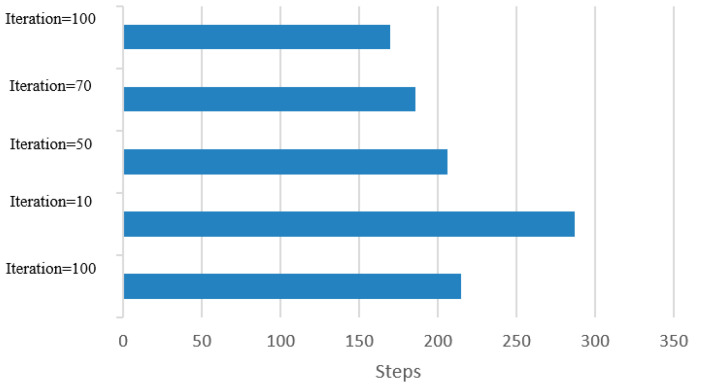
Sum of the costs per repetition to achieve the answer.

You can also see the statistics of the total number of peers examined in each iteration in Table 11 and Table 12. It can be seen that although the number of measurements performed in the proposed algorithm is initially high, with the implementation of the algorithm, the results are converged, and the number of analogies is reduced (Figure 10).

### 3.12. Compare the Proposed Method

**Figure 11:**
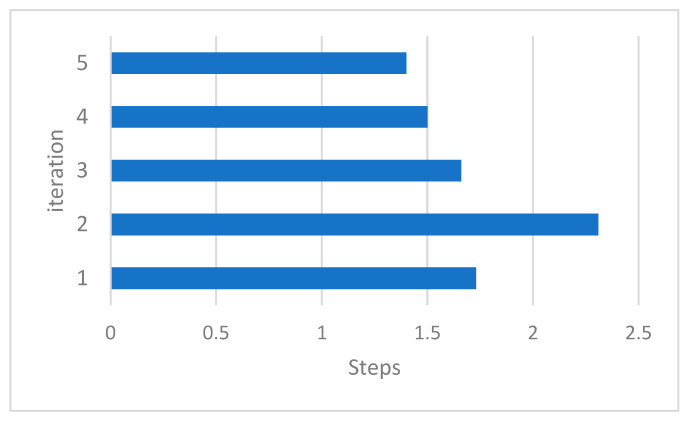
Average number of steps per repetition to achieve the answer.

**Figure 12:**
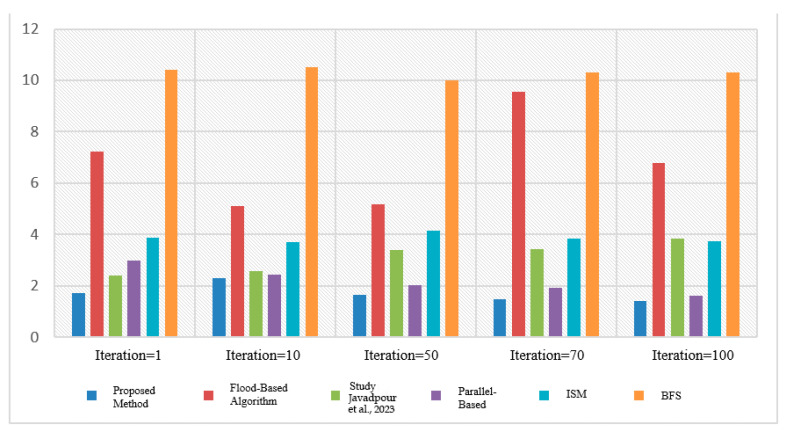
Comparison of the number of steps taken by the proposed method, flood, study [16], parallel diffusion algorithm and ISM method.

In this section, we look at various distinct strategies and compare them to the suggested algorithm. Flood technique, study [16], parallel diffusion algorithm, ISM intelligent search mechanism method, and Breath First Search (BFS) are examples of comparative approaches. The diagram below depicts the results of comparing various proposed methods. The purpose of the ISM technique is to limit the number of messages exchanged for each request as well as the number of nodes evaluated. They are above the present request and employ the two components of the Profile Mechanism and the Rating Mechanism to accomplish this. The structure of P2P networks with Super Peer (SP) is used in the proposed study technique [16], as it is in our study. The distinction is that in the study [16], a fuzzy logic-based system was employed to transmit the message. In this study, three P2P network input parameters are employed to generate output factor parameters: number of documents per peer (NDP), repetition percentage (RP), and scale of repetition per peer (SRP) (RF). In the parallel propagation approach, each node sends a request message to one of its neighbours, known as a walker, at random, and to save time, it can send numerous neighbours, known as k-walkers, instead of one. The number of messages increases linearly in relation to the number of nodes in this approach, and no consideration is given to the contents of the nodes used to transmit the message. Finally, in many existing systems, the first-level search approach is utilised, in which each node, after receiving the message, advertises the request to all neighbours excluding the sender, and then searches its local sources for the answer. If it discovers a response, it generates a QueryHit message and transmits it to the search node, with this packet following the precise path as the request message. This package provides information such as the quantity of relevant files and this node’s bandwidth, which will be useful in prioritizing the answer. This method, in addition to its simplicity, does not work well and does not use network resources well. Because each request consumes a large amount of network resources as it is transmitted to all links, low-bandwidth links can be problematic. In practice, to overcome this issue, a TTL (Time To Live) parameter is supplied for each request message, which determines the number of nodes that a request message can pass through, and this parameter decreases to zero after passing each node. This method finds a good percentage of responses for a large number of messages (Figure 11 and Figure 12).

**Figure 13:**
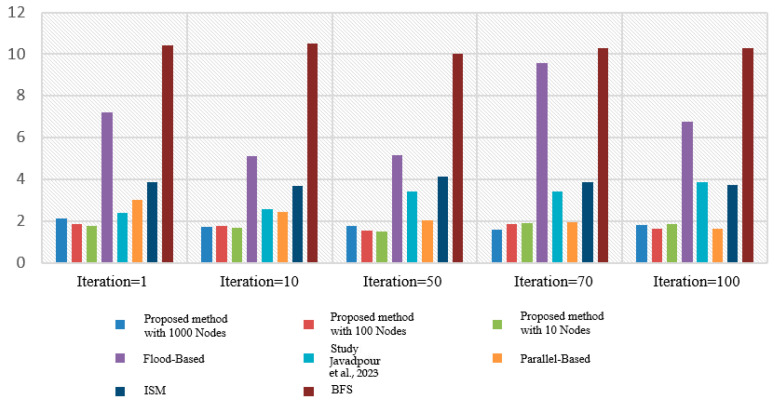
Comparison of the number of steps taken by the proposed method in the number of different nodes, flood, study [16], parallel diffusion algorithm and ISM method.

To evaluate the scalability of the proposed method, we conducted simulations for different network sizes, ranging from 10 nodes to 1000 nodes. The results of these simulations are presented in Figure 13 below. The figure compares the number of steps taken by the suggested technique with other approaches, including the flood algorithm, study [16], parallel diffusion algorithm, and the ISM method. Upon analyzing Figure 13, several key observations can be made. For a small number of nodes (e.g., 10 nodes), the suggested technique exhibits a relatively higher number of steps compared to other approaches, such as flood and the study [16]. As the number of nodes increases (e.g., 100 nodes and 1000 nodes), the number of steps taken by the suggested technique decreases significantly and converges with the number of steps taken by other approaches.

This convergence of the number of steps across different network sizes indicates that the proposed method demonstrates good scalability. It efficiently handles larger networks without compromising the effectiveness of query processing and node evaluation.

**Figure 14:**
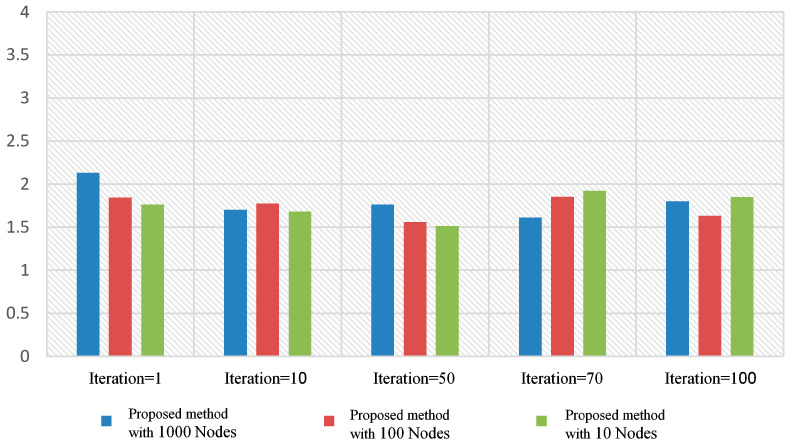
Comparing the number of steps taken by the proposed method with the number of different nodes.

To provide a more comprehensive analysis of the suggested method’s scalability, Figure 14 below presents a separate comparison of the number of steps performed by the proposed technique for each network size. The figure illustrates the efficiency of the suggested method in terms of the number of steps taken for 10 nodes, 100 nodes, and 1000 nodes separately. It shows how the number of steps decreases as the network size increases, demonstrating the scalability of the proposed algorithm. The evaluation of scalability through simulations reveals that the suggested technique adapts well to different network sizes, showcasing good scalability characteristics. While the number of steps may be slightly higher for smaller networks, it quickly converges as the network size increases. This demonstrates the efficiency and effectiveness of the proposed method in handling large-scale distributed collaborative applications. The analysis further supports the suitability of the suggested technique for peer-to-peer systems with varying numbers of nodes, making it a robust choice for real-world scenarios with dynamic and changing network environments.

### 3.13. Comparison of Execution Times

**Figure 15:**
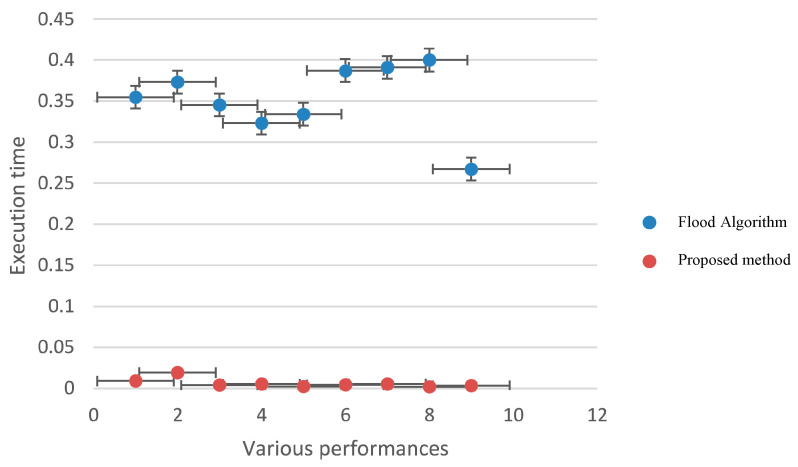
Comparison of execution time of the proposed algorithm and flood algorithm.

To further assess the performance of the suggested algorithm, we conducted a comprehensive comparison of its execution time with the flood algorithm. In order to obtain accurate results, we ran each program multiple times and measured the run time in different scenarios. The execution times of both algorithms were recorded, and the results are presented in Figure 15 below. The figure illustrates the comparison of the average execution times of the suggested algorithm and the flood algorithm. It provides a visual representation of the efficiency of both approaches in terms of run time.

Observations from the comparison indicate the following:

- The suggested algorithm exhibits competitive execution times compared to the flood algorithm. This demonstrates the effectiveness of the proposed intelligent search mechanism and profile-based node selection in optimizing query processing.
- As the network size and complexity increase, the advantage of the suggested algorithm becomes more pronounced, showing its ability to handle larger-scale distributed environments efficiently.
- The flood algorithm, while straightforward, may suffer from increased run time as it involves broadcasting requests to all nodes, leading to higher network resource utilization and potential bottlenecks.
- On the other hand, the suggested algorithm, leveraging intelligent node selection and limited message exchanges, demonstrates better scalability and resource management, contributing to its overall reduced execution time.

It is important to note that execution times may vary depending on factors such as network topology, the number of participating peers, and the nature of the queries. The presented comparison reflects the general performance trends of the algorithms under consideration. The comparison of execution times confirms that the suggested algorithm offers competitive performance and outperforms the flood algorithm in terms of run time. Its intelligent search mechanism and profile-based node selection contribute to enhanced efficiency and scalability, making it a promising choice for large-scale peer-to-peer distributed collaborative applications. Further experimentation and analysis may be conducted to explore the algorithm’s performance in diverse network scenarios and real-world environments.

## 4. Conclusions

Answering a query is one of the most difficult challenges in peer-to-peer databases, because receiving a complete answer is costly and time consuming. As a result, the use of approximation queries in these systems was contemplated. The primary goal of this research was to develop an intelligent system for answering approximate set-value inquiries. In this paper, the particle optimization algorithm is used to improve intelligence, and, contrary to previous work, it is attempted not to use sampling in the proposed method; because even with the best sampling methods with the highest accuracy, there is still the possibility of error, and there is never a guarantee that the desired accuracy will be achieved. However, in approximate queries, the most important goal is to achieve the user’s desired accuracy. In reality, the accuracy of sampling procedures is determined by various factors. The results of the studies reveal that the suggested method has improved in terms of the number of queries issued and the number of peers examined, as well as its execution time, which is significantly faster than the flood approach. Answering a query is one of the most difficult challenges in peer-to-peer databases, because receiving a complete answer is costly and time consuming. As a result, the use of approximation queries in these systems was considered. The primary goal of this research was to develop an intelligent system for answering approximate set-value queries. In this paper, the particle optimization algorithm is used to improve intelligence, and, contrary to previous work, it is attempted not to use sampling in the proposed method, as even with the best sampling methods with the highest accuracy, there is still the possibility of error, and there is never a guarantee that the desired accuracy will be achieved.

## Figures and Tables

**Table 2:** The details parameters for simulating a peer-to-peer system.

HDD	Bandwidth	RAM	Number of CPU	Resources
5 TB	10 GB	32 GB	8	MAX
500 GB	2 GB	2 GB	2	MIN
100 GB	1 GB	256 MB	1	Step

**Table 3:** Profiles of cluster heads in different clusters.

Storage	Bandwidth	RAM	CPU	Index Number	Cluster
23	9	31	7	4	Cluster 1
43	6	19	7	8	Cluster 2
48	9	18	4	3	Cluster 3
11	8	25	4	7	Cluster 4
14	10	16	7	5	Cluster 5
12	6	19	3	10	Cluster 6
14	10	16	7	5	Cluster 7
48	9	18	4	3	Cluster 8
9	7	3	5	14	Cluster 9
15	8	13	6	1	Cluster 10
48	9	18	4	3	Cluster 11

**Table 4:** Index table for cluster heads 1 to 10.

Cluster Head	Number of Indexes Stored				
1	5	23	25	30	-
2	4	9	19	26	27
3	7	9	15	19	32
4	6	7	16	25	26
5	5	7	14	25	-
6	15	17	20	29	30
7	2	17	19	28	-
8	3	9	23	-	-
9	1	12	16	19	-
10	2	15	29	30	-

**Table 5:** Index table for cluster heads 1 to 10.

Cluster Head	Number of Indexes Stored				
1	5	23	25	30	35
2	4	9	19	26	27
3	7	9	15	19	32
4	6	7	35	25	26
5	5	7	14	25	-
6	15	17	20	29	30
7	2	17	19	28	-
8	3	9	23	-	-
9	1	12	16	19	35
10	2	15	29	30	-

**Table 6:** Parameters used in PSO algorithm.

Value	Description	Parameter
1024	Initial number of peers	n
32	number of peers in each cluster	eachCluster

**Table 7:** Number of communication links between each cluster head to other clusters.

Group 1	Group 2	Group 3	Group 4	Group 5	Group 6	Group 7
4	2	7	6	6	8	5

**Table 8:** Comparison of the overall number of peers examined.

First Iteration	Tenth Iteration	Fiftieth Iteration	Seventieth Iteration	Hundredth Iteration
617	632	480	4101	3290

**Table 9:** Numerical value of the average cost per iteration to obtain the answer.

First Iteration	Tenth Iteration	Fiftieth Iteration	Seventieth Iteration	Hundredth Iteration
4.93	5.05	3.84	32.80	26.32

**Table 10:** The number of scrolls necessary in the proposed algorithm to obtain the answer.

First Iteration	Tenth Iteration	Fiftieth Iteration	Hundredth Iteration
3	4	4	3
4	4	2	2
4	3	3	3
5	4	4	3
4	3	4	3
3	3	2	2
3	3	2	3
4	4	3	2

**Table 11:** Comparison of the total number of peers examined.

First Iteration	Tenth Iteration	Fiftieth Iteration	Seventieth Iteration	Hundredth Iteration
215	287	206	186	170

**Table 12:** Numerical value of the average cost per repetition to achieve the answer.

First Iteration	Tenth iteration	Fiftieth Iteration	Seventieth Iteration	Hundredth Iteration
1.73	2.31	1.66	1.5	1.4

## Data Availability

The data that support the findings of this study are available from the corresponding author, upon reasonable request.
